# The Methods and Experiments of Shape Measurement for Off-Axis Conic Aspheric Surface

**DOI:** 10.3390/ma13092101

**Published:** 2020-05-01

**Authors:** Shijie Li, Jin Zhang, Weiguo Liu, Haifeng Liang, Yi Xie, Xiaoqin Li

**Affiliations:** 1 Shaanxi Province Key Laboratory of Thin Film Technology and Optical Test, Xi’an Technological University, Xi’an 710021, China; zhangjin@xatu.edu.cn (J.Z.); wgliu@163.com (W.L.); hfliang2004@163.com (H.L.); 2 School of Optoelectronic Engineering, Xi’an Technological University, Xi’an 710021, China; 3 Display Technology Department, Luoyang Institute of Electro-optical Equipment, AVIC, Luoyang 471009, China; xyi613@163.com; 4 Library of Xi’an Technological University, Xi’an Technological University, Xi’an 710021, China; rileyhome923@126.com

**Keywords:** off-axis conic surface, shape accuracy, auto-collimation, single CGH, hybrid compensation

## Abstract

The off-axis conic aspheric surface is widely used as a component in modern optical systems. It is critical for this kind of surface to obtain the real accuracy of the shape during optical processing. As is widely known, the null test is an effective method to measure the shape accuracy with high precision. Therefore, three shape measurement methods of null test including auto-collimation, single computer-generated hologram (CGH), and hybrid compensation are presented in detail in this research. Although the various methods have their own advantages and disadvantages, all methods need a special auxiliary component to accomplish the measurement. In the paper, an off-axis paraboloid (OAP) was chosen to be measured using the three methods along with auxiliary components of their own and it was shown that the experimental results involved in peak-to-valley (PV), root-mean-square (RMS), and shape distribution from three methods were consistent. As a result, the correctness and effectiveness of these three measurement methods were confirmed, which are very useful in engineering.

## 1. Introduction

Off-axis aspheric optical elements are often used in modern optical systems such as off-axis TMA (Three Mirror Anastigmatic), telescope (e.g., GMT (Giant Magellan Telescope), TMT (Thirty Meter Teloscope), E-ELT (European Extremely Large Telescope)), and so on [1,2]. In order to achieve high quality, the off-axis aspheric optical element has become the focus of research in optical manufacturing. To evaluate the quality of off-axis aspheric surface, the related optical measurement technique is necessary to gain the shape accuracy, which is the key parameter of the optical element. Usually, interferometry is commonly used to test the shape accuracy of the flat and the spherical optical elements. However, until now, there has been no unified method for aspheric surfaces, especially off-axis aspheric surfaces, to test their profile with a nano-precision.

To measure the shape error of the off-axis aspheric surface at a high accuracy, researchers have offered several useful methods [3,4,5,6,7,8,9,10,11,12,13]. Wang Xiao-kun tested an off-axis ellipsoid mirror with sub-aperture stitching interferometry and applied least-squares fitting to process the test data, resulting in a 1.275λ of PV and 0.113λ of RMS [4]. Similarly, Yongfu Wen tested an off-axis hyperboloid mirror with off-axis annular sub-aperture stitching interferometry and obtained the results by a complex calculation [5]. Obviously, this sub-aperture stitching technology requires more measuring time, and the more complex data processing method is not a null test technique [4,5,6]. Jan Burke used a flat mirror as the aiding element to detect a 90° off-axis paraboloid mirror, which obtained the results of PV = 343 nm and RMS = 50 nm. Although this method belongs to the auto-collimation measurement method, the adjustment processing is difficult [7]. Similarly, Ki-Beom Ahn used a spherical convex reference mirror as the aiding element to detect the ellipsoid mirror, which was the secondary mirror of the Giant Magellan Telescope (GMT) [8]. Due to the diameter of this ellipsoid (up to 1.06 m), the aperture size of the spherical convex reference mirror would reach 0.99 m, leading to serious difficulties in the fabrication of this aiding element. Additionally, the adjustment processing is tough. As a null test technique, the computer generated hologram (CGH) method is a suitable compensator for off-axis aspheric surface measurement [9,10,11]. M. M. Talha measured a freeform surface with the CGH method with a result of PV = 0.0479λ [12]. Similarly, Li Fa-zhi used CGH as the aiding element to measure an off-axis high order aspheric surface [13].

When the aperture of the off-axis aspheric surface is up to the meter level and the asphericity rises to the millimeter level, the CGH method will not be suitable because the difficulty and cost of CGH fabrication increase dramatically. In this instance, the application of the two aiding elements, fold mirror together with GGH, could be a good choice. J. H. Burge used this method to measure the segment of primary mirror of the GMT, which was an off-axis aspheric surface with an aperture size of 8.4 m [14]. In the same way, Chang Jin Oh measured an off-axis paraboloid with a 4.2 m aperture diameter and 9 mm asphericity [15].

In order to obtain accurate measurement results of the off-axis conic aspheric surface, three null test methods are introduced in Section 2. After that, an off-axis paraboloid (OAP) was chosen to be tested in Section 3. The aiding elements from the three methods were designed and fabricated respectively to measure the OAP. Finally, the correctness of three methods were mutually cross-checked by their experimental results.

## 2. The Shape Measurement Methods

Interferometry is frequently reckoned as an effective tool to test the shape accuracy of the optical surface; however, it is limited to directly measuring flat or spherical surfaces. Due to the inherent aberration of the off-axis aspheric surfaces, an auxiliary optical element is required for the null test. Moreover, the aiding element changes with the variation of the measurement method.

**Figure 1:**
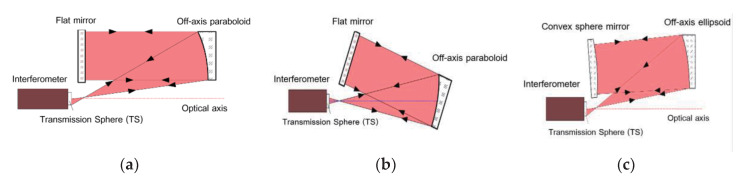
Schematic diagram of auto-collimation: (**a**) off-axis paraboloid located at off-axis; (**b**) off-axis paraboloid located at on-axis; (**c**) off-axis ellipsoid located at off-axis.

Due to having perfect image points, a conic aspheric surface such as paraboloid, ellipsoid, and hyperboloid can be measured using the classical null test method of auto-collimation. Within this method, a flat mirror (for paraboloid) or a spherical mirror (for ellipsoid or hyperboloid) is required as the aiding optical element. As shown in Figure 1, two optical layouts for measuring an off-axis paraboloid with flat mirror and one layout for measuring an off-axis ellipsoid with a convex sphere mirror are illustrated.

### 2.1. Auto-Collimation Method

The auto-collimation method is inexpensive and does not need a complex design or a long time to prepare. To make this method simple and useful, the aperture and the F-number of the aiding element should be considered. As shown in Equation (1), there are three errors occurring in the test results of this method.

(1) $$Error_{total}=Error_{TS}+Error_{aiding}+Error_{aspheric}$$

In this equation, *Error_total_* is the total error of the measurement system, *Error_TS_* is the error of the TS (transmission sphere, a standard lens used on the interferometer), and *Error_aiding_* is the error of the aided element (such as flat mirror or convex sphere mirror). *Error_aspheric_* is the error of the off-axis aspheric under testing. Usually, the quality of TS is so high that *Error_TS_* is very small. If the *Error_aiding_* is also much smaller than *Error_aspheric_*, the test result *Error_total_* is approximately equal to *Error_aspheric_*. Otherwise, when the *Error_aiding_* is not small enough, we can subtract *Error_aiding_* from *Error_total_* to obtain *Error_aspheric_* after calibrating the *Error_aiding_*. However, for off-axis aspheric surfaces, it is difficult to adjust all the components to the correct location. If there exists alignment error, there will be unavoidable misalignment errors such as astigmatism and coma in the test results. As a result, the adjustment turns into the most difficult process within the auto-collimation method.

The limitation of the auto-collimation method is attributed to the surface type and aperture of the off-axis aspheric under testing. As the aperture of the aiding element should be larger than the off-axis aspheric, this is a difficult and expensive mission if the aperture is up to meter level.

### 2.2. Single Computer Generated Hologram (CGH) Method

**Figure 2:**
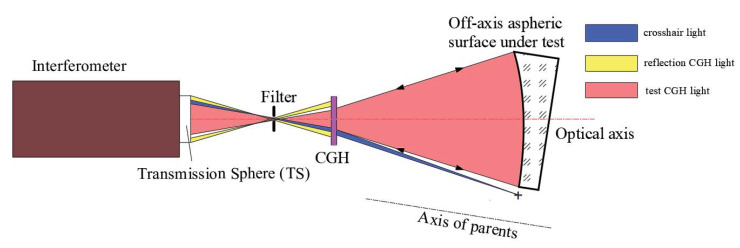
Schematic diagram of single computer generated hologram (CGH) method for off-axis aspheric surface.

As a diffractive optical element, CGH can produce any shape wavefronts [9]. Figure 2 shows the principle of this method. The interferometer, CGH, and off-axis aspheric under testing are all on the same axis, so the aberration that should be compensated will be reduced [16].

In Figure 2, the output wavefront after TS is the convergent sphere wavefront. However, the CGH changes the sphere wavefront into the off-axis aspheric wavefront to match the theoretical shape of the off-axis aspheric surface under testing, where this off-axis aspherical wavefront vertically illuminates on the surface. After that, the wavefront with the information of the off-axis aspheric surface is reflected again into CGH via the same path it comes from, and ultimately back to the interferometer to form interference fringes, from which we eventually gain the test results. The test result with the single CGH method is also involved in several errors, as shown in Equation (1), by replacing *Error_aiding_* with *Error_CGH_*. Before the CGH basement reaches the precision requirement, it should be processed to achieve a high quality in surface shape and parallelism, and then processed by photoetching to ensure the quality of the CGH [17]. As a result, the *Error_CGH_* is too small to have an effect on the testing results.

The CGH includes three parts: the test CGH, reflection CGH, and crosshair CGH. The test CGH is the transmission diffraction part (the red part in Figure 2), which creates the same wavefront of the off-axis aspheric surface under testing. When the off-axis aspheric surface is different, the corresponding CGH varies according to the customized design. Reflection CGH is used to align the interferometer and CGH (the yellow part in Figure 2), and crosshair CGH is applied to align the CGH and off-axis aspheric under testing (the blue part in Figure 2). 

**Figure 3:**
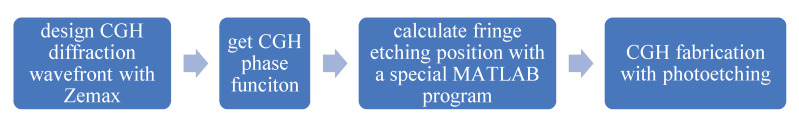
CGH design process.

Within the single CGH method, the CGH design is the most important part. The geometry parameters in Figure 2 determine the size of the CGH and the aberration to be compensated. As it is a key parameter in the single CGH method, the aberration should be compensated by the CGH diffraction wavefront that decides the measurement accuracy and can be designed with Zemax (Version June 9, 2009). After obtaining the CGH phase function, the fringe etching position can be calculated with a special MATLAB program (R2016b). The design process is shown in Figure 3.

The disadvantage of the single CGH method lies in the minimum fringe spacing and the size of the whole CGH. When the minimum fringe spacing is less than the ability of photoetching, this customized CGH cannot be fabricated successfully.

### 2.3. Hybrid Compensation Method

**Figure 4:**
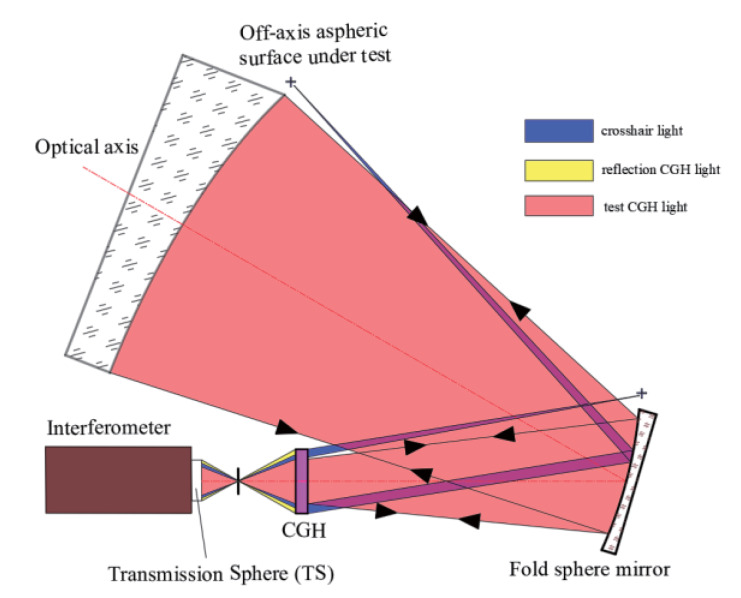
Schematic diagram of the hybrid compensation method for off-axis aspheric surface.

Hybrid compensation is also an interference measurement technique to solve the shape measurement problem of the off-axis aspheric surface. When the aberration is larger than what a single CGH cannot be compensated, hybrid compensation is a suitable choice. In Figure 4, a fold sphere mirror was used to compensate most of the low-order aberration and a CGH was applied to compensate the residual aberration, which is still a null test system [18]. The function of each element in Figure 4 is similar to that illustrated in Figure 2 of Section 2.2.

The test result of hybrid compensation includes four parts, as seen in Equation (2),

(2) $$Error_{total}=Error_{TS}+Error_{\mathrm{CGH}}+Error_{sphere}+Error_{aspheric}$$

In this equation, the definition of each sub-term is similar to that in Equation (1). The *Error_CGH_* from the CGH is very difficult to measure directly and can be estimated by an indirect method [19,20]. Similar to the first two methods (Section 2.1 and Section 2.2), when *Error_TS_*, *Error_CGH_*, and *Error_sphere_* are all small enough, the test result *Error_total_* is approximately equal to *Error_aspheric_*. Otherwise, the error of these elements should be separately calibrated and then subtracted from the test result to obtain the error of the off-axis aspheric under testing.

In this measurement system, the fold sphere and CGH should be designed. According to laboratory conditions, the fold sphere is chosen by selecting the main parameters of the curvature radius and aperture. Meanwhile, the location position and fold angle of this fold sphere mirror should consider whether it will disturb the measurement. After choosing the fold sphere mirror and determining the geometrical parameters of its measurement system, the CGH can be designed. The CGH design processing is the same as that of the single CGH method, which is shown in Figure 3.

As shown in Figure 4, this hybrid compensation measurement system possesses four elements of the folded optical axis, resulting in a complex system that is characterized with huge difficulty in adjusting. To reduce the difficulty, the customized CGH is divided into multiple parts. In addition to testing the CGH, others are used to align between different elements such as the interferometer and CGH, the fold sphere and CGH, and the aspheric and CGH.

The proposed three measurement methods that can all test the shape accuracy of conic off-axis aspheric surface belong to the null test. The auto-collimation method, which is the simplest technique with the simplest aiding element, is possessed of stronger generality. Single CGH and hybrid compensation both need customized CGH, so they have less generality. The customized CGH, which is a diffractive element, needs long and expensive preparation. However, these two methods can measure more surface types with the assistance of adjustment marks, resulting in a wider range of application.

## 3. Experiments

To verify the correctness of these methods, we used three methods to measure the same conic off-axis aspheric surface. According to the conditions of our laboratory, an off-axis paraboloid (OAP) was chosen and its parameters are shown in Table 1.

### 3.1. Auto-Collimation Method

**Figure 5:**
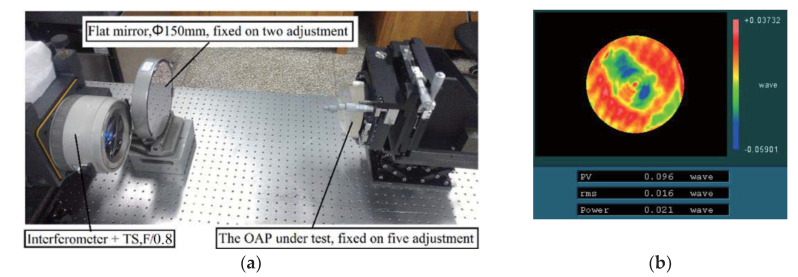
(**a**) Photo of auto-collimation for this OAP; (**b**) the error of flat mirror (PV = 0.096λ).

First, we used the classical auto-collimation method to measure this OAP. The result can be seen as the real shape distribution of this OAP. According to Figure 1a, this OAP is located on the off-axis position. Meanwhile, a Φ150 mm flat mirror was chosen as the aiding optical element. A 6-inch Zygo interferometer (Middlefield, CT, USA) with an F/0.8 transmission sphere (TS) was used. The quality of this TS was 1/10λ (PV), and the shape error of the flat mirror measured via interferometry was about 0.1λ (PV). The corresponding error distribution is seen in Figure 5. According to Equation (1), the testing results can be reckoned as the error of OAP because the errors of TS and flat mirror were both far less than that of the OAP.

Due to its location on an off-axis position in Figure 5, the OAP was adjusted to guarantee the position accuracy by a five-dimension adjusting tool. In this measurement system, the adjustment processing is very difficult because of a lack of alignment marks. Coma, astigmatism, and power aberration always exist because of the misalignment of different optical elements. Therefore, adjusting experience is critical. After repeating the adjusting until there is almost no coma and astigmatism, a credible testing result will be obtained.

### 3.2. Single CGH Method

**Figure 6:**
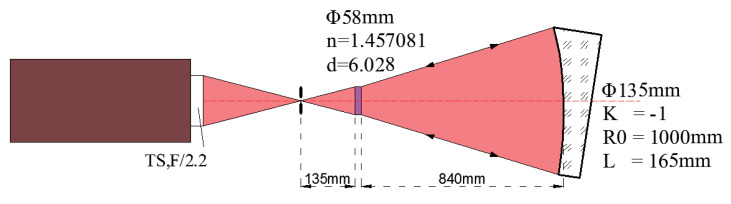
The geometry parameters of measuring this OAP with a single CGH.

**Figure 7:**
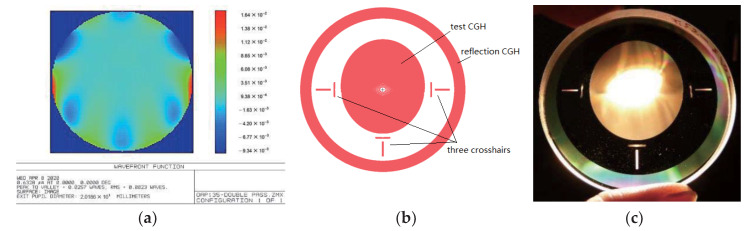
(**a**) Simulated residual wavefront of the single CGH method (test CGH) in Zemax (PV = 0.0257λ, RMS = 0.0023λ); (**b**) simulation pattern of the customized CGH; (**c**) CGH photograph.

Second, we tested this OAP with the single CGH method. Depending on the conditions of our laboratory, silica (Φ58 mm, n = 1.457081 and d = 6.028 mm) was chosen as the CGH basement. Before processing the CGH, this silica basement was processed by IBF (ion beam figuring) to guarantee that its shape error was less than 5 nm (RMS) and its wedge angle was less than 10 s. The geometry parameters of this measurement system are defined in Figure 6, from which the designing result of a customized single CGH could be conducted and is demonstrated in Figure 7. The CGH wavefront was designed using Zemax software and the etching map was generated by the specialized MATLAB program (Figure 3).

**Figure 8:**
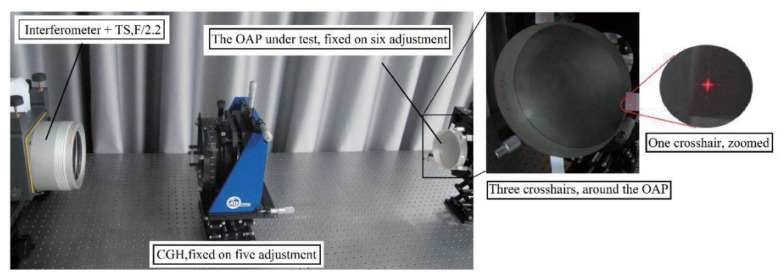
Photo of measuring the OAP with the single CGH method.

After obtaining the design result of the customized CGH seen in Figure 7b, photoetching was used to fabricate the CGH with high manufacturing precision. Even so, CGH still had some errors and the real error of CGH was very difficult to calibrate directly. According to the corresponding estimation method of the CGH error, the total CGH error was about 7.8 nm (RMS), which is a negligible value compared with the error of the OAP [19,20]. The customized single CGH includes three parts: the test CGH (the inner part), reflection CGH (the outer part), and three crosshairs (three orthogonal rectangular couples), which are attributed to different functions. The test CGH, which is a transmission CGH using first order diffraction, was fabricated by a grating groove depth of 692.2 nm. It can be seen that the test CGH is the most important because it was adopted to measure the shape of the OAP. As shown in Figure 7a, the residual wavefront of test CGH was only 0.0257λ (PV) and 0.0023λ (RMS). With a grating groove depth of 158.2 nm, the reflecting CGH, which made use of the third order diffraction, was used to align the interferometer and CGH. With a grating groove depth of 692.2 nm, three crosshairs, which were transmission CGHs using first order diffraction, were applied to align the OAP and CGH. The combined CGH was fabricated by photoetching, as shown in Figure 7c. The measurement experiment is shown in Figure 8 with an obvious crosshair. Relying on these crosshairs, it was easy to adjust the various optical elements to its own correct position.

### 3.3. Hybrid Compensation Method

**Figure 9:**
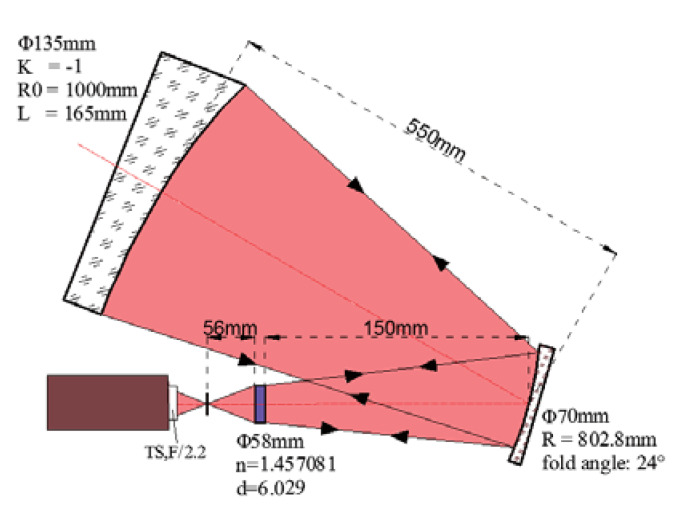
The geometry parameters of measuring this OAP via the hybrid compensation method.

**Figure 10:**
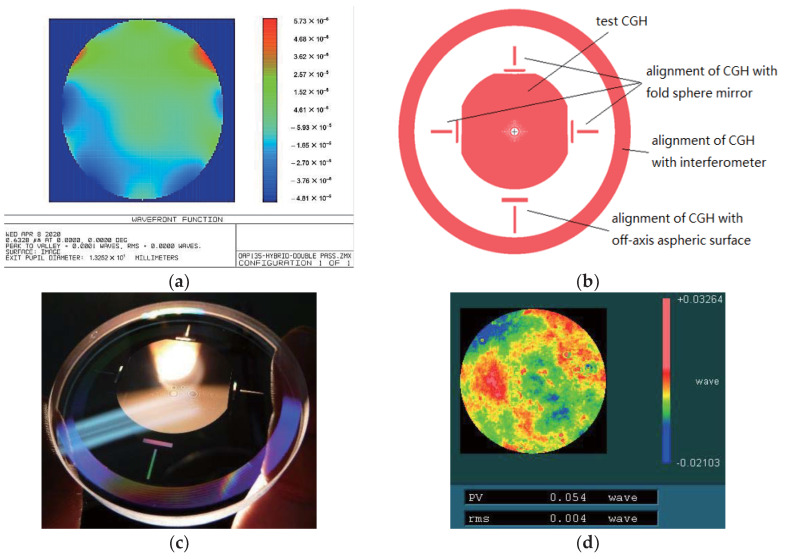
(**a**) Simulated residual wavefront of the hybrid compensation method (test CGH) in Zemax (PV = 0.0001λ); (**b**) simulation pattern of the customized CGH for this hybrid compensation system; (**c**) CGH photograph; (**d**) error of the fold sphere mirror (PV = 0.054λ).

Third, we tested the OAP using the hybrid compensation method. In the same way, the CGH basement (silica) and the fold sphere mirror were chosen according to our laboratory, and the related geometry parameters are shown in Figure 9. Ultimately, the design result of the customized CGH for the hybrid compensation system is illustrated in Figure 10.

Another multiple combined CGH was designed and produced. The accuracy of the CGH basement was the same as that of the basement in Section 3.2. As shown in Figure 10, the multiple combined CGH was involved in four parts such as the test CGH, alignment CGH and so on. Three parts were transmission CGH, except the part aligning CGH with the interferometer. The test CGH and alignment CGH with the fold sphere mirror both adopted the first order diffraction with the same grating groove depth of 692.2 nm. However, other parts adopted the third order diffraction with the corresponding groove depth of 158.2 nm. The residual wavefront of the test CGH was only 0.0001λ (PV), as shown in Figure 10a. At the same time, the error of the fold sphere mirror, which was measured via interferometry, was about 0.05λ (PV), far less than the error of this OAP.

**Figure 11:**
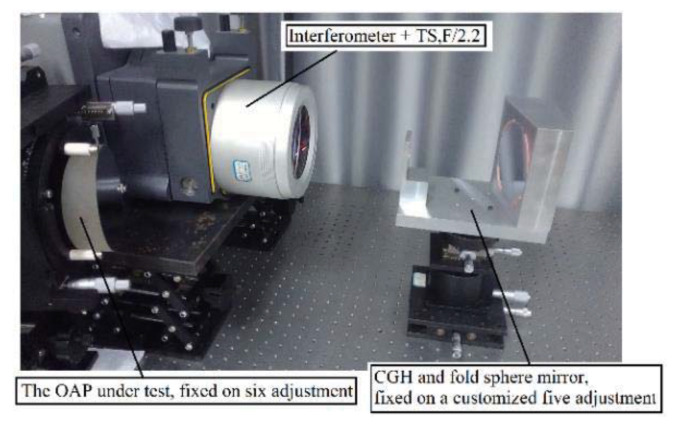
Photo of measuring this OAP via hybrid compensation (with customized fixture).

During the measuring process, the customized CGH was placed close to the fold sphere mirror by a customized fixture, with which the relative position between the CGH and fold sphere could be guaranteed, as presented in Figure 11. At the same time, the customized fixture was mounted on a five-dimensional adjuster so that they would be regarded as the whole during the adjusting process. After repeatedly adjusting, the position error of each element can be reduced to a negligible value. Meanwhile, the errors of the fold sphere mirror and CGH were far less than the error of OAP, so the testing result can be regarded as the shape error distribution of the OAP.

### 3.4. Experimental Results with These Three Methods

**Figure 12:**
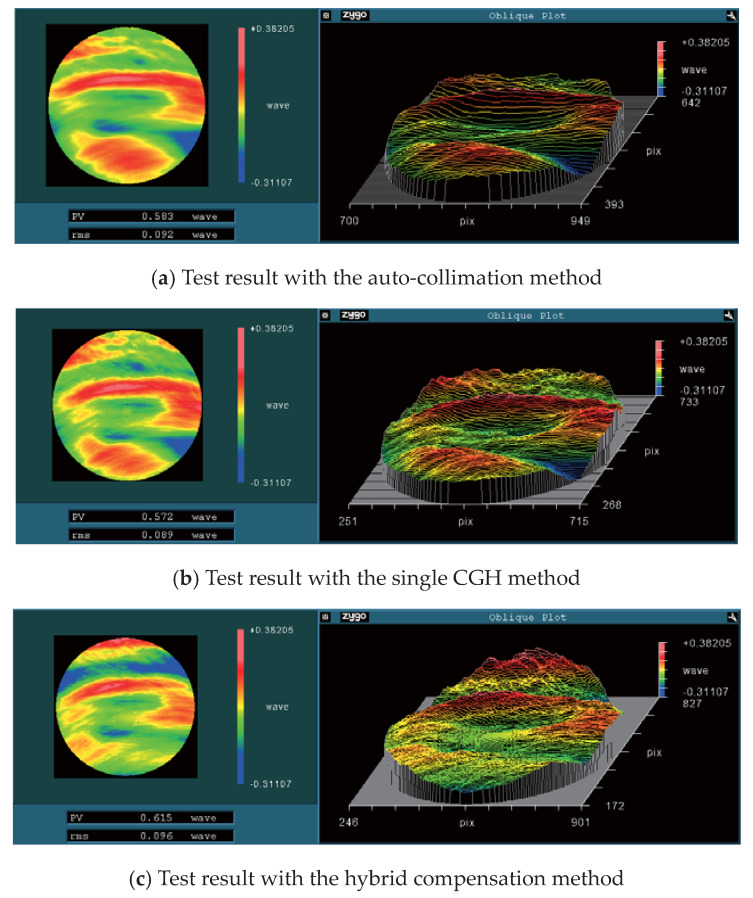
The test result of Φ135 mm OAP: (**a**) auto-collimation; (**b**) single CGH; (**c**) hybrid compensation.

This OAP was measured using three methods, respectively. After the measured data were simply processed in MetroPro software (V9.1.1) [21] to remove piston, tilt, and power, the experimental results were obtained and are shown in Figure 12. 

The resulting value of auto-collimation method as PV = 0.583λ and RMS = 0.092λ, the resulting value of the single CGH method as PV = 0.572λ and RMS = 0.089λ, and the resulting value of the hybrid compensation method was PV = 0.615λ and RMS = 0.096λ. The testing results with different methods were nearly the same, although there was a slight difference on the shape distribution, which was mainly caused by the mapping distortion of CGH. Furthermore, there were a few small residual adjustment errors, some of which were particularly induced by the angle adjustment in the hybrid compensation method, as it is a delicate process. Hence, a more accurate alignment technique is still being investigated.

## 4. Conclusions

Shape accuracy, as a key parameter of the off-axis conic aspheric surface, has an important effect on the imaging quality of the optical system, so it is necessary to guarantee the shape is accurate during the measuring process. In this study, we provided three methods to test the shape accuracy of the off-axis conic aspheric surface with high precision. The first was auto-collimation, which belongs to a classical and simple method with a requirement of a flat mirror or a sphere mirror as the aiding element. However, this method has some limitations to the surface type and the aperture of off-axis surface under testing. The second was the single CGH, which is widely used as an effective method with alignment marks to reduce the adjustment difficulty. However, this method requires a customized CGH, which varies with changes in off-axis aspheric surfaces, making it an expensive option. With the need of a fold sphere mirror and a customized CGH, the third is hybrid compensation, which belongs to a more complex measurement technology. The method has a stronger aberration compensation ability and is more suitable for the off-axis aspheric surface of large aperture and large asphericity. Unfortunately, its adjustment is more difficult. Therefore, we recommend not using this method unless absolutely necessary.

In this study, an OAP was measured via these three methods. By the means of auto-collimation, a Φ150 mm flat mirror was used as the aiding element, and the result was PV = 0.583λ and RMS = 0.092λ; by the means of the single CGH, a customized CGH was designed and fabricated, and the result was PV = 0.572λ and RMS = 0.089λ; by the means of hybrid compensation, a fold sphere mirror was chosen, and another customized CGH was also designed and fabricated where the result was PV = 0.615λ and RMS = 0.096λ. These three measurement methods brought forth approximate results, in the meantime, the shape distributions were also close to each other, which proves that these three measurement methods can all obtain comparatively accurate testing results. Furthermore, these three methods can also cross-check the correctness of each other and other available methods.

## Figures and Tables

**Table 1:** The parameters of the chosen off-axis paraboloid (OAP).

Type of Aspheric	Aperture	Conic	Vertex Radius of Curvature	Off-Axis Distance
Off-axis paraboloid	135 mm	−1	1000 mm	165 mm
